# Propranolol as a Novel Therapeutic Approach for Post-Stroke Anxiety: A Clinical Review and Future Directions

**DOI:** 10.7759/cureus.76381

**Published:** 2024-12-25

**Authors:** Abdelrahman Abdelhameed, Aly Abouslima, Aya Sayed Hamdy Ghanem, Salma Ahmed, Rajia Islam, Shahida Sultana, Mohamed A Khoudir

**Affiliations:** 1 Neuroscience, Barking, Havering and Redbridge University Hospitals NHS Trust, London, GBR; 2 Acute Medicine, University Hospitals Birmingham NHS Foundation Trust, Birmingham, GBR; 3 Diagnostic Radiology, Minia University Hospital, Minia, EGY; 4 Cardiology, Faculty of Medicine, Mansoura University, Mansoura, EGY

**Keywords:** narrative review, post-stroke anxiety, propranolol therapy, psychiatric agitation, stroke

## Abstract

Stroke remains a leading cause of death globally, with survivors facing both physical and psychological challenges. While acute stroke treatment has improved, many patients develop post-stroke anxiety (PSA), particularly generalized anxiety disorder. PSA represents a significant clinical challenge as most stroke survivors suffer from it. Current management includes lifestyle changes, behavioral therapy, and medications like selective serotonin reuptake inhibitors (SSRIs). Propranolol, a non-selective beta-blocker, has shown promise in treating anxiety disorders by targeting physical symptoms through its effects on the sympathetic nervous system. Its dual benefits in managing both cardiovascular and psychiatric symptoms make it particularly relevant for stroke patients. We aim to evaluate the therapeutic potential of propranolol in managing PSA and to establish a framework for future clinical studies comparing its effectiveness with SSRIs in stroke patients. Propranolol presents a potentially advantageous therapeutic option for post-stroke patients due to its multifaceted clinical benefits. Its dual mechanism of action addresses both neuropsychiatric symptoms and cardiovascular complications, offering simultaneous management of anxiety, cardiac arrhythmias, and hypertension, conditions that frequently coexist in stroke patients. We recommend high-quality randomized trials to estimate the efficiency of propranolol in comparison to other interventions and to estimate the cost-effectiveness while taking into consideration the associated risk.

## Introduction and background

A stroke is a medical emergency classically characterized as a neurological deficit due to an acute focal vascular injury of the central nervous system. The types of vascular injuries include cerebral infarction and intracerebral hemorrhage (ICH) [1]. Stroke is the second leading cause of death worldwide, with approximately 5.5 million deaths annually; early stroke intervention is critical, with every minute delay causing significant neuron loss [2]. Stroke is classified into three major types: ischemic stroke, hemorrhagic stroke, and sub-arachnoid stroke. Ischemic stroke is the most common type and represents about 87% of all cases in the United States [3]. According to a previous analytical study, the risk of post-stroke anxiety (PSA) is higher in the cases of hemorrhagic stroke [4]

Stroke is influenced by a combination of risk factors, with hypertension being the most common. Approximately 64% of stroke patients have a history of hypertension. Age is also another critical risk factor, as the risk of stroke approximately doubles with each decade of life after the age of 55 [5]. It is noteworthy that women face a higher risk of stroke, as pregnancy increases risk three-fold through elevated estrogen and blood coagulation, oral contraceptives raise risk by 1.7-2.3 times, and post-menopausal estrogen decline promotes atherosclerosis and reduces vasodilation, partially due to factors associated with female sex hormones [6,7]. The clinical presentation of stroke varies depending on the site of the focal injury within the CNS but commonly includes sudden onset of muscle weakness, numbness, fascial drooping, and impairments in cognition and coordination [8,9].

In the last two decades, the treatment of acute stroke has improved, so the mortality rate related to stroke has decreased, and the rate of survival with moderate disability has increased [10]. Conventionally, studies on post-stroke functional abnormalities mainly focused on motor and sensory disabilities, language disorders, and challenges in daily activities. However, long-term follow-up studies of stroke survivors have shown that a considerable number of individuals also suffer from psychological distress and various psychiatric disorders [10,11]. The psychiatric conditions associated with stroke include mood changes such as depression, anxiety disorders, post-traumatic stress disorder (PTSD), and aggressive and apathetic personality changes [12].

Anxiety can be categorized into four main subtypes: phobic anxiety, social anxiety, panic disorders, and generalized anxiety disorder (GAD), which is the most prevalent after a stroke [13]. GAD is characterized by chronic anxiety and excessive, uncontrollable worry lasting for at least six months, with lifetime prevalence rates of 4-7% [14-16].

The management of PSA includes lifestyle modification, cognitive behavioral therapy, and drug therapy. Previous small pilot trials investigating the use of relaxation therapies to alleviate PSA have demonstrated hopeful results and have demonstrated that various relaxation techniques can effectively reduce anxiety symptoms in stroke survivors. These early findings support the potential therapeutic value of relaxation-based interventions, particularly during early stroke recovery when anxiety levels tend to peak [16,17].

The drug therapy for PSA is still not guided. However, the drugs currently used are also effective. It includes antidepressants such as selective serotonin reuptake inhibitors (SSRIs) and noradrenaline reuptake inhibitors. A meta-analysis involving 175 individuals with PSA and depression showed that paroxetine and buspirone reduce the score of anxiety [18]. Other drugs like benzodiazepines and buspirone are also used. Another systematic review and meta-analysis demonstrated that SSRIs are effective for the improvement of PSA and depression [19]. Furthermore, GAD related to stroke may be prevented by the use of escitalopram (SSRI) or problem-solving therapy [20].

Also, propranolol is now used in a limited way to treat anxiety disorders. Propranolol is a non-selective beta-blocker discovered for the first time in the 1960s by Sir James Black. It competes with catecholamines at the level of ß1,2-adrenoreceptor, which is why propranolol inhibits the sympathetic effect of these receptors [21]. Propranolol was initially made to treat angina, but then propranolol proved great efficacy in the treatment of cardiovascular diseases like hypertension, dysrhythmias, and myocardial infarction [22].

As atrial fibrillation is another risk factor for the development of stroke, propranolol would show high effectiveness for the maintenance of a normal heart rate and resolving the symptoms of arrhythmia, resulting in better control for the stroke in addition to the psychiatric effects [20]. Furthermore, it was discovered that propranolol is effective in the management of several non-cardiovascular diseases such as migraine, portal hypertension, hyperthyroidism, pheochromocytoma, essential tremors, and psychiatric conditions like PTSD and anxiety [23,24]. As mentioned above, the first use of propranolol in anxiety was in the 1960s [25]. It was found that propranolol is effective in managing the symptoms of hyperactivity and hyperarousal, which are commonly associated with anxiety disorders. It is also used to suppress the physical symptoms associated with GAD that are caused by noradrenergic stimulation, such as tachycardia, palpitations, sweating, and tremors. Also, propranolol can reduce preoperative anxiety [22,26]. In this review, we aim to shed light on the use of propranolol for controlling the anxiety, agitation, and aggressiveness that result after the development of stroke; we also aim to give future recommendations for clinical professionals to perform studies investigating the effectiveness in comparison to SSRIs without having any study design restrictions as we will obtain all the available evidence.

## Review

Results

Prevalence of Post-Stroke Agitation/Irritability/Anxiety

Post-stroke neuropsychiatric symptoms, particularly agitation, irritability, and anxiety, demonstrate notably high prevalence rates across multiple epidemiological studies.

In a meta-analysis including 44 studies with 5760 stroke patients conducted in 2012 by Campbell et al., they found that the overall pooled estimated anxiety in stroke patients was 18% according to clinical interviews, while the estimated result assessed with a rating scale showed a higher percentage (25%) [27]. The study concluded the frequent occurrence of anxiety, whether by interviewing or using the scale, and shed light on the importance of further investigation for the risk of anxiety and the negative impacts on patients. Similar results were obtained by Schöttke and Giabbiconi when investigating PSA among 289 stroke patients, showing that the prevalence of anxiety was 20.4%; their study linked that the post-stroke comorbidities are the main result for both post-stroke depression (PSD) and PSA [28]. In a more recent systematic review and meta-analysis that was conducted in 2018, it was reported that the prevalence of anxiety following stroke was 29.3% in the first year following stroke, with a strong recommendation for routine screening post-stroke [29]. This study showed an increase in the prevalence of PSA in comparison to the previous meta-analysis.

Yao et al. investigated the relationship between PSA and the COVID-19 outbreak, specifically in elderly patients; they found that the prevalence of anxiety was 30.1%, which showed no different results from the other studies [30]. Although the study gave extra conclusions showing that COVID-19 and age were not considered risk factors in the development of post-stoke anxiety, aligning with other studies [27,30]. Another study that was made during the COVID-19 pandemic showed a prevalence of 32% for anxiety following a stroke, which is also similar to other literature [31]. The substantial prevalence of PSA, affecting approximately one-third of stroke survivors, represents a significant clinical concern that warrants heightened attention in post-stroke care.

Noradrenergic System and Its Role in PSA

The noradrenergic system plays a crucial role in modulating the fear response and anxiety. Its primary components include noradrenergic neurons, norepinephrine (NE), and adrenergic receptors. NE is considered the key neurotransmitter within the system, as it regulates arousal and facilitates adaptation to internal and external stress [32,33].

NE is synthesized by noradrenergic nuclei located mainly in the locus coeruleus (LC) within the brainstem. LC plays a main role in the regulation of autonomic activity and arousal. The receptors of the noradrenergic system are metabotropic and classified into α-adrenoreceptors and β-adrenoreceptors [25].

The activation of α1-adrenoreceptors and β-adrenoreceptors by the NE results in the excitation of the cells that are innervated by the noradrenergic system. In comparison, the activation of α2-adrenoreceptors inhibits the innervated cells [34,35].

The LC innervates the amygdala, which is a key component of the limbic system. The amygdala plays a critical role in mediating fear and anxiety responses to threatening stimuli. [36,37]. The amygdala mainly expresses the excitatory α1-adrenoreceptors, so the overall noradrenergic influence on the amygdala is excitatory [38]. Stimulation of the LC increases anxiety levels by enhancing the excitatory pathway from the LC to the amygdala [39,40].

Anxiety is associated with an activated autonomic nervous system, which is explained by different changes such as irritability, palpitation, tachycardia, shallow breathing, and excessive sweating. Also, these changes are associated with augmented LC activity that enhances sympathetic activities and inhibits parasympathetic discharges [41].

PSA disorders have been relatively under-researched compared to PSD. The primary symptoms of PSA include persistent excessive worry and difficulty managing these concerns. According to the Diagnostic and Statistical Manual of Mental Disorders, the diagnosis of PSA requires the presence of three or more additional symptoms, such as restlessness, fatigue, impaired concentration, irritability, muscle tension, or insomnia, alongside the core features [42].

Effectiveness of Propranolol in PSA

Propranolol has been used in the past decades for the treatment of situational anxiety, as in many healthy people without any previous history of hypertension or arrhythmia, to reduce the physical symptoms of anxiety [43]. A previous meta-analysis investigated the effects of beta-blockers on the treatment of anxiety and found limited evidence supporting their efficacy in anxiety treatment, yet their prescription in primary care continues to increase. This highlights the need to investigate the clinical decision-making patterns behind beta-blocker prescriptions for anxiety in primary care [44]. Previous research suggests propranolol's potential extends beyond acute treatment to preventive applications in chronically stressed individuals. Studies have demonstrated that propranolol not only reduces anxiety-related behaviors but also enhances stress resilience for subsequent stressors [45].

Cost-Effectiveness of Treatment with Propranolol

Anxiety disorders are considered one of the most prevalent mental health conditions, imposing a significant burden on individuals and society. Therefore, it is important to evaluate the cost-effectiveness of various interventions. However, recent studies addressing economic evaluations are still insufficient [46]. While SSRIs are considered the first-line treatment for anxiety disorders, their annual cost is still higher when compared to propranolol. The recommended duration of treatment for anxiety disorders varies widely, ranging from three to six months to one to two years or longer, depending on the severity of the condition and the patient’s response to therapy [47]. According to a study in 2005, the annual cost of generic SSRIs for managing anxiety disorders fell between $158 and $332 for an average of 10.14 prescriptions annually, reflecting adjustments for non-adherence, therapy changes, and dosage adjustments [48].

On the other hand, according to data provided by the Canadian Agency for Drugs and Technologies in Health, propranolol doses in the management of anxiety range from 40 mg to 160 mg per day, depending on the severity of symptoms and individual response. The approximate cost of a generic preparation of propranolol is $3.675 for 30 tablets of 40 mg (0.122 for tablet). Based on dosage, the monthly cost ranges from $3.675 at a minimum dose to $14.70 at the maximum dose, translating to an annual cost between $44.10 and $176.40 [49]. The cost comparison demonstrates propranolol's significant economic advantage over SSRIs. Moreover, while SSRI prices have shown consistent annual increases of 5-7.5% in recent years, with an upward trend expected to continue, propranolol remains a more cost-effective alternative for anxiety management.

Discussion

A high prevalence of PSA was documented in several epidemiological studies, affecting one-third of post-stroke patients, underscoring the threat that PSA represents for stroke survivors, which requires accurate interventions to reduce the burden resulting from the condition and to improve the quality of life for the associated patients. Moreover, early diagnosis may help in the alleviation of the cases and prevent their worsening.

Several points should be noted when estimating the prevalence, as the assessment methodology affects the final estimate. A psychologist could do the assessment through an interview or by fixed scale. This also may raise questions on which method is more reliable [50]. The increased prevalence in the most recent studies is primarily a reflection of the improvement in the screening methods with better detection or could possibly detect an actual increase in PSA cases in recent years.

Till now, the exact cause of PSA has not been detected. Some studies have indicated that strokes affecting the right frontal and temporal lobes of the brain may increase the likelihood of developing anxiety after a stroke [51,52]. Also, a study demonstrated that patients with ischemic stroke affecting the thalamus are frequently associated with PSD and PSA [53]. Conversely, a meta-analysis that included five studies and involved 1054 stroke survivors has detected that there was no association between the site of the lesion and the anxiety after stroke [53]. In addition, no association was detected between sex and age. This finding suggests the heterogenous nature of PSA that may be a result of neuroanatomical disruption or imbalance or psychosocial factors, which requires clinical attention for the management of anxiety-associated stroke cases, as they should consider both the neuroanatomical and psychosocial factors in addition to the lesion location.

Gu et al., in 2024, suggest that anxiety following a stroke might be a result of the dizziness that resulted from a stroke, which may explain the high incidence of PSD and PSA [54]. The bidirectional relationship between dizziness and psychological symptoms represents a significant clinical consideration in post-stroke care. The presence of dizziness appears to initiate a complex feedback loop where physical discomfort and functional limitations can trigger psychological distress, mainly manifesting as anxiety and depression [55]. Concurrently, these manifestations may exacerbate the perception and impact of dizziness symptoms, potentially creating a self-perpetuating cycle of physical and mental distress [54].

PSA may be classified into early-onset anxiety and late-onset anxiety. It is believed that early onset occurs within the first few weeks after the stroke and is related to the immediate effect of stroke on brain structures such as the limbic system. In contrast, late-onset anxiety is related to chronic psychological stress due to long-term disability. Also, early-onset anxiety is more frequently linked to a history of prior psychiatric disorders compared to late-onset anxiety. This has led to the hypothesis that early-onset anxiety may represent a recurrence or exacerbation of pre-existing GAD following a stroke [42,56].

PSA is closely associated with psychiatric conditions and psychological factors. Previous studies have reported a strong correlation between PSA and PSD [57-60]. Also, patients with a history of anxiety, depression, and dementia are more susceptible to PSA [61]. The differential predictability of PSA and PSD based on lifetime history reveals distinct pathophysiological mechanisms, offering valuable insights for clinical practice [28]. While PSA demonstrates strong predictability based on pre-existing anxiety history, suggesting the significant role of psychological vulnerability factors, PSD shows no such correlation with lifetime depression, indicating that stroke-specific neurobiological factors may play a more dominant role in its development [28].

The existing evidence for propranolol in post-stroke agitation and anxiety presents significant limitations that warrant careful consideration. Most available data comes from cohort studies, lacking the robustness of large-scale randomized controlled trials. A notable challenge in the current literature is the absence of standardized definitions for post-stroke agitation and anxiety, compounded by variable assessment timing across acute and chronic phases. The heterogeneity in measurement tools and outcome measures further complicates the synthesis of available evidence. The use of propranolol in clinical practice requires careful attention to the drug activity within the body and its effects on the cardiovascular system, especially when used to treat anxiety. Since propranolol takes time to show its anti-anxiety effects, healthcare providers must plan the treatment carefully to ensure optimal patient outcomes.

## Conclusions

PSA affects approximately one-third of stroke survivors, representing a significant clinical challenge. The noradrenergic system is crucial in PSA pathophysiology, providing a theoretical basis for propranolol's therapeutic potential. Propranolol offers a unique therapeutic advantage in post-stroke care through its concurrent management of anxiety symptoms and cardiovascular complications, providing an integrated treatment approach for patients presenting with both conditions. We can see that propranolol offers a cost-effective alternative to SSRIs as it is lower in cost than most of the available SSRIs and demonstrates promise in anxiety management; current evidence supporting its use in PSA remains limited. The complex nature of PSA, involving both neurobiological and psychosocial factors, necessitates a comprehensive treatment approach. We recommend large-scale randomized controlled trials in order to establish the efficacy and safety profile in PSA treatment
